# Case of Rupture of the Uterus

**Published:** 1855-04

**Authors:** C. Milford Crandall

**Affiliations:** Belfast, N. Y.


ART. IV.—*Case of Rupture of the Uterus.* By C. MILFORD CRANDALL, M. D., Belfast, N. Y.

January 17, 1855, called 9 o'clock, A. M., to Mrs. Roach, aged 35 years, confined with her eighth child, four of which died in utero. Learned from the patient that she was attacked with the pains of labor about three hours before. On examination found the os uteri dilating, presentation natural and every thing favorable for a natural labor. In about one hour after, the labor in the meantime continuing very regular, the membranes burst and rather more than an ordinary quantity of liquor amnii evacuated.

Examination now showed the fœtal head engaging in the superior strait. Pains now became active but not unusually severe. During perhaps the fifth or sixth pain after evacuation of the waters, patient said: "Dr., I thought I was going to get right through, but the child has taken a wonderful leap and gone back; I could not have been hurt worse had I been struck with an axe; O, such a severe cramp in my side, my breath will stop," &c., &c., with a great many similar expressions indicating the most severe distress.

I was now surprised to find that the head of the child had receded entirely beyond reach of the fingers' touch.

The agony of the patient continuing so severe, I changed her position slightly, gave her a dose of morphine, and ordered a warm fomentation of spirits of camphor to the side.

A new difficulty arose: uterine action was suspended; I ordered some warm drinks and a warm pediluvium, to no effect.

The true and formidable nature of the case now presented itself to my mind—*rupture of the uterus.* This truth, the character of the friends and the decided aversion of the patient to anything like manual interference (having lost a sister a few months previous immediately after the operation of turning) made it necessary for me not to proceed further without counsel. I therefore sent for Dr. Charles, of Angelica, and Dr. Allen, of New Hudson. The latter arrived in due time. On his arrival I proceeded to introduce my hand into the uterus, which I found rid of all its contents excepting one arm of the child, which had not escaped into the peritoneal cavity. Following the arm through the rupture, which was nearly longitudinal, in the left side of the body of the uterus toward the fundus, till reaching the body, I sought for and secured the feet, brought it by their means back into the uterus and per vaginam into the world. The child, from appearance, had been dead some time.

The previous labors of Mrs. R. had been characterized by being very severe and lingering.

The general symptoms were, as would be expected from so extensive an injury. There was no great change in the pulse previous to delivery, but it became very irregular and feeble a few hours after. The respiration from the time the accident occurred was hurried, labored and painful; the countenance soon became anxious, the eyes sunken; the abdomen very tender to the touch and much swollen, even some swollen before delivery.

After delivery the patient, notwithstanding the administration of narcotics, continued in severe agony until very near her death, which occurred thirty-four hours after delivery.

